# Preclinical and clinical progress for HDAC as a putative target for epigenetic remodeling and functionality of immune cells

**DOI:** 10.7150/ijbs.62001

**Published:** 2021-08-03

**Authors:** Sijia Zhang, Lingjun Zhan, Xue Li, Zhenhong Yang, Yumin Luo, Haiping Zhao

**Affiliations:** 1 Institute of Cerebrovascular Disease Research, Xuanwu Hospital of Capital Medical University, Beijing, China.; 2 Beijing Geriatric Medical Research Center and National Clinical Research Center for Geriatric Disorders, Beijing, China.; 3 Beijing Institute for Brain Disorders, Capital Medical University, Beijing, China.; 4 Institute of Laboratory Animal Science, Chinese Academy of Medical Sciences, Beijing, China.

**Keywords:** Epigenetics, HDAC, neutrophils, mast cells, macrophage, dendritic cells, lymphocyte, microglia, astrocyte

## Abstract

Genetic changes are difficult to reverse; thus, epigenetic aberrations, including changes in DNA methylation, histone modifications, and noncoding RNAs, with potential reversibility, have attracted attention as pharmaceutical targets. The current paradigm is that histone deacetylases (HDACs) regulate gene expression via deacetylation of histone and nonhistone proteins or by forming corepressor complexes with transcription factors. The emergence of epigenetic tools related to HDACs can be used as diagnostic and therapeutic markers. HDAC inhibitors that block specific or a series of HDACs have proven to be a powerful therapeutic treatment for immune-related diseases. Here, we summarize the various roles of HDACs and HDAC inhibitors in the development and function of innate and adaptive immune cells and their implications for various diseases and therapies.

## Introduction

Although the immune system is generally defined as a defense system against pathogens, it is increasingly recognized as playing a crucial role in tissue repair after injury. The plasticity of immune cells has extensive implications in the pathogenesis and resolution of cancers, autoimmune diseases, chronic inflammatory disorders, and metabolic disorders. With highly coordinated and dynamic alterations in chromatin structure and gene expression programs, immune cells can exquisitely identify, respond, and produce a rapid and augmented response to pathogens or tissue injury in immune-related diseases. Increasing evidence has shown that immune cell plasticity can be altered by epigenetic modulation.

Epigenetics is typically defined as heritable changes in genome function due to DNA and histone modifications, nucleosome remodeling, and expression of noncoding RNAs, as opposed to the genetic code, which distinctly influence several aspects of cell physiology and pathology 1, 2. The high plasticity of epigenetic modifications due to environmental variability provides a novel area for therapeutic intervention. The epigenetic regulation of gene expression requires a balance in the acetylation/deacetylation of histone and nonhistone proteins controlled by histone deacetylases (HDACs) and histone acetyltransferases (HATs) 3, 4. Recently, several HDAC-based diagnostic biomarkers and drugs have received regulatory approval or entered clinical practice or both. For example, HDAC inhibitors (HDACi) are being evaluated for the treatment of hematological malignancies and solid tumors. Here, we review recent advances in our understanding of classical HDACs as potential targets for epigenetic regulation of distinct immune cell types in both preclinical studies and clinical drug trials in inflammatory and autoimmune diseases and discuss the potential translational implications and challenges.

## Classes of Histone deacetylases (HDACs) and HDAC inhibitors (HDACi)

### Classes of Histone deacetylases (HDACs)

To date, 18 types of mammalian HDAC isoforms have been identified. Based on their structure, function, and homology of accessory domains to histone deacetylases, HDACs are categorized into four classes: Class I (HDAC 1, 2, 3, and 8); Class II (HDAC 4, 5, 6, 7, 9, and 10); Class III (SIRT 1, 2, 3, 4, 5, 6, and 7), and Class IV (HDAC11). Classes I, II, and IV HDACs are zinc-dependent and also termed classical HDACs, while class III HDACs, termed non-classical HDACs, are NAD(+)-dependent 5. Whether these HDAC isoforms are redundant or specific for controlling gene expression programs in eukaryotic cells remains unclear. Pharmacological studies using selective HDAC inhibitors (HDACIs) and HDAC-knockout mice have revealed the specificity of individual HDACs in the development of various diseases 6.

### Regulation of deacetylation by HDACs

Acetylation is a major post-translational protein modification that introduces an acetyl functional group into amino acids, such as lysine (the residue), to yield an acetate ester bond, neutralizing its positive charge. Presently, the acetylome encompasses nearly 40,000 unique protein acetylation sites, as classified in the PhosphoSitePlus database 7. Regulation of protein functions by acetylation occurs in multiple ways: by affecting protein stability, activity, localization, and interaction with other proteins or DNA 8. HATs and HDACs are the enzymes involved in this modification and are thought to act as critical gene silencers or activators. HATs increase the acetylation of histones, presenting a less condensed DNA segment, which is prone to transcription. HDACs are evolutionarily conserved enzymes that remove acetyl groups from histones and nonhistone proteins such as hormone receptors, signaling proteins, chaperones, transcription factors, and DNA damage response proteins, with functional consequences on chromatin remodeling and gene expression profiles 9. HDACs regulate target genes via ε-amino group deacetylation of histone lysine residues, thus promoting DNA condensation, and therefore, transcriptional silencing. Moreover, certain HDACs are not lysine deacetylases, but rather lysine fatty-acid deacylases 10; others process non-protein substrates by acting as polyamine deacetylases 11.

### Classes of HDAC inhibitors (HDACi)

HDACi modulate gene expression by interacting with HDACs. These processes are target-specific reactions among various types of HDACis and HDACs 12. HDACis are classified according to their chemical structures as follows: short-chain fatty acids (sodium butyrate [NaBu] and valproic acid [VPA]); benzamides (entinostat [MS-275] and mocetinostat [MGCD0103]); hydroxamic acids (panobinostat [LBH-589], belinostat (PXD101), vorinostat (SAHA), scriptaid (ST), and trichostatin-A [TSA]); cyclic peptides (romidepsin [FK228]), and miscellaneous compounds (depudecin) 13 (Table 1).

### Pharmacokinetic and pharmacodynamic evaluation of HDACi treatment

To improve the clinical potential of HDACIs, a prodrug strategy has been utilized to improve the *in vivo* pharmacokinetic and pharmacodynamic performances of HDACIs, such as hydroxamic acid, carboxylic acid, thiol, benzamide, anticancer drug, and carrier-conjugated prodrugs 14. Early research focused on developing the pharmacokinetic/pharmacodynamic tools necessary for novel HDACi studies in canine cancer clinical setting 15. A positive correlation was found between VPA dose and histone hyperacetylation in peripheral blood mononuclear cells 15. In examining compound stability and cellular permeability, PTG-0861, a novel HDAC6-selective inhibitor, revealed a promising *in vitro* pharmacokinetic profile 16. Besides, structure-activity relationship studies have been performed to verify the influence of the linker on the biological profile of the compounds 17. Recently, a novel photo-activated HDACi was identified to cage metacept-3 15, with pharmacokinetic activation characteristics and biological properties that may make it suitable for evaluation as a novel coating for targeted drug eluting balloon catheter angioplasty interventions 18. These studies could help enhance the stability, water solubility, lipophilicity, oral bioavailability, tumor cell selectivity, and further structural modification of HDACIs with high potential as drug candidates.

### Clinical investigation of HDACi

HDACs are considered potential next-generation therapeutics, given the promising potential of their broad HDAC inhibition or HDAC isoform-selective inhibition for multiple diseases. An emblematic example is the prospect of HDACi in treating cancer, which is attracting extensive attention. HDACIs have exhibited potency against various cancer types, and four of them: SAHA, FK228, PXD101, and LBH-589, have been approved by the US FDA (Foods and Drugs Authority) for cancer treatment 14. SAHA and FK228 have proven to be effective in treating cutaneous T-cell lymphoma (CTCL) and have already been applied clinically, while chidamide has been approved as the first oral subtype-selective HDACi in China. SAHA and FK228 were both demonstrated to have significant and sustainable single-agent activity with acceptable safety profiles in patients with CTCL in phase IIb multicenter trials 19-21. PXD101, a non-specific HDAC inhibitor (pan-HDACi), was proven to be effective and well-tolerated in patients with peripheral T-cell lymphoma (PTCL) or cutaneous T-cell lymphoma (CTCL) in a multicenter study 22. The safety and activity of LBH-589, a nonselective HDACi, in relapsed/refractory Hodgkin's lymphoma were also proven in a multicenter, phase II trial, and showed a significant reduction in tumor size 23. Some specific HDACi are currently undergoing clinical trials. Chidamide is the first oral subtype-selective HDACI (HDAC1,2,3,10) approved in China and the first HDACI of the benzamide class approved for treating relapsed and refractory PTCL 24. In a phase I study, KA2507, a potent and selective inhibitor of HDAC6, showed selective target engagement, no significant toxicities, and prolonged disease stabilization in a subset of patients with refractory solid tumors 25.

HDACis have also been trialed in other diseases. VPA is a widely prescribed antiepileptic drug, and a longitudinal observational study showed that VPA efficacy for focal or generalized seizures is superior to that of mixed-type seizures 26. VPA has been used in treating bipolar disorder since the 1990s 27, and has shown positive results in spinal muscular atrophy in cohort studies 28. The successful trial of HDACis, which are currently available for clinical use in the treatment of cancers, hints at the existence of the cures for various human diseases such as epilepsy through epigenetic regulation.

## Preclinical studies of HDACs and HDACi in different types of immune cells

Immune cells in the circulatory system can be divided into innate immune cells (neutrophils, eosinophils, basophils, mast cells, macrophages, dendritic cells, and natural killer cells) and adaptive immune cells (T and B lymphocytes). The immune system of the central nervous system, which is relatively independent, primarily consists of two types of glial cells, microglia and astrocytes. These glial cells intimately participate in immune responses in multiple CNS diseases.

### HDAC and neutrophils

Neutrophils, eosinophils, basophils, and mast cells are crucial in host defense against pathogens. Neutrophils, which are key in the first line of defense, extensively exist in the circulatory system and are deemed indispensable for HDACs. Neutrophils act as effector and regulatory cells to defend against pathogens, and perform functions such as phagocytosis, generating antimicrobial enzymes and toxic factors, releasing inflammatory cytokines, and discharging nuclear DNA and chromatin proteins which form neutrophil extracellular traps (NETs) 29. The mechanism of NET generation (NETosis) is based on the special cell death process of neutrophils. After liberation, NETs may block invaders and facilitate the restoration of homeostasis. Neutrophils are classically defined as terminally differentiated, short-lived cells; however, they can be long-lived with phenotypic plasticity. As regards their high plasticity, neutrophils can acquire an anti-inflammatory N1 or proinflammatory N2 phenotype. Besides, a subset of neutrophils transdifferentiate into neutrophil-dendritic cell hybrids (PMN-DCs), a group of cells with both neutrophil and dendritic cell properties during inflammation.

Emerging evidence has supported the essential role of HDACs in regulating the proliferation, differentiation, and apoptosis of neutrophils. Knockdown of HDAC1 or HDACi (SAHA and MS-275) reduced cell expansion, increased apoptosis of neutrophil progenitor cells, and decreased the percentage of mature neutrophils 30. HDAC11 is a recently discovered member of the HDAC family. Among myeloid cells, neutrophils are considered the largest producers of HDAC11. The HDAC11 yield increases throughout the differentiation and maturation of neutrophils. However, the migratory and phagocytic capacities of inflammatory cytokines were elevated in neutrophils from mice lacking HDAC11, which were found to be more vulnerable to lipopolysaccharide (LPS)-induced sepsis, granulocytic expansion, and splenomegaly due to increased extramedullary hematopoiesis 31. This implies that HDAC11 is a vital factor that influences neutrophil function. However, the links between HDAC11 and some noteworthy matters, such as neutrophil NETosis, neutrophil activation, and antagonistic neutrophil populations, remain largely unknown. Recently, researchers found that histone acetylation has prompted NETosis in human neutrophils via induction by NOX-dependent or NOX-independent pathway agonists. HDACi belinostat and panobinostat, which cause neutropenia in clinical practice, could shift neutrophil NETosis to apoptosis 32, 33. Another study showed that NETs may influence the autoimmune response in systemic lupus erythematosus (SLE) by providing extra autoantigens 34. The paper argued that histones in NETs produced by SLE-derived neutrophils showed higher levels of acetylation and methylation. Furthermore, pan-HDACi TSA treatment performed before NETosis induction was found to stimulate hyperacetylation of histones in NETs, thus enhancing their ability to activate macrophages, eventually prompting a shorter exposure period to NET antigens 35.

In contrast, HDACi can inhibit disease progression by depressing neutrophil activity and migration. SAHA, HDAC6-shRNAs treatment, or Tubacin (selective HDAC6-inhibitor) could reduce neutrophil activities, thus regressing cystic fibrosis-lung disease 36. HDACi NaBu was demonstrated to mediate the therapeutic effect on the LPS-induced endometritis mouse model by reducing neutrophil granulocyte concentration and TNF-α and IL-1β levels 37. The HDAC1-3 inhibitor MS-275 attenuated neutrophil infiltration into the lungs in a model of cigarette smoke-exposed mice 38. A class I HDACi, CI-994, suppressed the accumulation of neutrophils and expression of inflammatory cytokines in a mouse model of spinal cord injury (SCI), thus contributing to promoting functional recovery, especially during the early stages of SCI 39. Our group demonstrated HDAC2-mediated neutrophil infiltration and N1/N2 phenotypic shift following ischemic stroke 40. In summary, HDAC and HDACi play necessary roles in the differentiation, NETosis, immune responses, and migration of neutrophils under normal and pathological conditions. However, the effect of HDAC on different diseases is inconsistent, which is related to the pathological mechanism by which neutrophils influence the disease pathogenesis.

### HDAC and eosinophils

Eosinophils exhibit intense proinflammatory effects in some pathological situations. Besides, eosinophils can contribute to immunoregulation and antiviral activity. Eosinophils lineage-restricted progenitors (EoP), the progenitors of eosinophils, are reasoned to be downstream of the granulocyte/macrophage progenitors (GMP) in mice and the common myeloid progenitor (CMP) or the upstream pre-CMP progenitor in humans 41, 42. Various environmentally adaptive phenotypes of mature eosinophils are located in the mucosae of multiple organs, such as the gastrointestinal tract and lungs. Functional heterogeneity is discovered not only among eosinophils that reside in different tissues, but also between residential eosinophils under homeostasis and eosinophils recruited after immune responses 43, 44.

**Figure 1:**
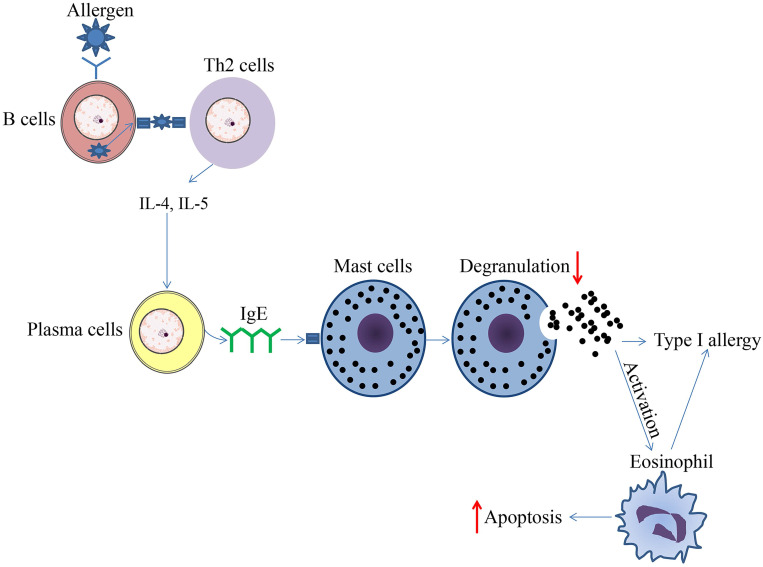
Schematic diagram of effect of HDAC inhibitors on immune cells during type 1 allergy. The upward arrows represent the promotion effect by HDAC inhibitors and the downward arrows represent the suppression effect by HDAC inhibitors.

The epigenetic regulation by HDAC and HDACi has been shown to be involved in the control of eosinophil function under normal homeostatic and pathogenic conditions in various tissues. Lung eosinophilia is a hallmark of asthma, and eosinophils are reasoned to affect the pathogenesis of allergic inflammatory diseases significantly. RGFP966 was recently shown to reduce HDAC3 expression and HDAC3 activities; eosinophil and mast cell recruitment, goblet cell proliferation, and inflammatory cytokine levels were decreased, triggering the alleviation of allergic and inflammatory responses in an OVA-induced allergic rhinitis mouse model 45. A previous study showed that short chain fatty acids (SCFAs) attenuated human eosinophils at several functional levels, including (1) adhesion to the endothelium, (2) migration, and (3) survival 46. *In vivo*, butyrate ameliorated allergen-induced airway and lung eosinophilia, reduced type 2 cytokine levels in bronchial fluid, and improved airway hyperresponsiveness in mice. *In vitro* and *in vivo* findings highlight the importance of SCFAs, especially butyrate, as a promising therapeutic agent for allergic inflammatory diseases 46. In a mouse asthma model, TSA reduced allergen-induced airway inflammation and airway constriction partly by reducing the eosinophils numbers in bronchoalveolar lavage fluid 47-49. Moreover, TSA promoted the apoptosis of human eosinophils, indicating the potential therapeutic effect of HDACi on eosinophil-related diseases such as chronic obstructive pulmonary disease (COPD) and asthma (Figure 1) 50. HDAC1 could be a molecularly targeted treatment for specific granule deficiency with homozygous mutation for two amino acids, arginine (R247) and serine (S248), which were deleted in the basic leucine zipper domain of C/EBPε (ΔRS) 51. In summary, the present research mainly demonstrated the abrogation of HDACi on eosinophil function, suggesting a promising therapeutic effect of HDACi on eosinophil-related inflammatory disorders.

### HDAC and mast cells

Mast cells are multifunctional. They perform different tasks when encountering different immune triggers, circumstances, and cytokine stimulation. Mast cells (MCs) are enduring pluripotent leukocytes that mainly reside in the mucosa, airways, and skin, while a small number are found in the perivascular tissue of large blood vessels and between cardiomyocytes 52. They are components of the innate immune system, characterized by their granules that contain inflammatory mediators such as histamine and tryptase, and can produce many more inflammatory cytokines, such as leukotrienes and prostaglandins 53. Until now, two subtypes of MCs have been recognized: MCTCs, which are typically found in the mucosa and can produce tryptase and chymase, and MCTs, which contain tryptase but not chymase. MCTs are mainly present in connective tissue 53.

The role of HDACi in the inhibition of MC-mediated inflammatory responses has been demonstrated in previous studies; for example, SAHA and MGCD0103 were able to inhibit antigen-induced histamine release in rat peritoneal mast cells (Figure 1) 54. When binding to IgE, bone marrow-derived mast cells (BMMCs) activate and produce higher levels of IL-4, IL-6, TNF-α, and IL-13. This process could be reversed to some extent by TSA pretreatment. The results showed that with TSA pretreatment, cytokines secreted by mast cells were altered, while both FcεRI amount and mast cell degranulation declined (Figure 1). After TSA treatment, I-κBα transcription increased while phospho-RelA levels and NF-κB activation were decreased. TSA treatment may play far more roles than previously thought. It could also impact MC experienced non-IgE stimuli, and continuous TSA exposure could accelerate mast cell apoptosis (Figure 1). Finally, an ovalbumin-induced food allergy mice model induced showed that TSA treatment remarkably relieved allergic diarrhea and decreased mast cell activation 55. In summary, these findings demonstrate that histone acetylation may be an important factor affecting epigenetic modification during mast cell activation, and exposure to dietary substances may affect the epigenetic regulation of mast cells. Current studies have demonstrated the anti-allergy effect on mast cells, indicating the potential future application of HDACi in related fields.

### HDAC and macrophages

Macrophages and neutrophils mount early immune protection against intracellular pathogens; however, neutrophils are short-lived cells, and removal of apoptotic cells by resident macrophages is a key event in resolving inflammation and tissue repair. Monocytes and macrophages, the most plastic cells of the hematopoietic system, are derived from bone marrow progenitors that continually proliferate and shed their progeny in the bloodstream as promonocytes. Circulating monocytes then migrate into almost all tissues, where they differentiate into macrophages under local growth factors, proinflammatory cytokines, and microbial products, and display great functional diversity and affect development, homeostasis, tissue repair, and immunity 56. Although macrophages are activated in various microenvironments, they are recognized to exist in two distinct phenotypes: 1) the proinflammatory M1 macrophages, which are polarized by microbicidal stimuli including LPS or Th1 cytokines such as IFN-γ, and could respond to the stimuli by triggering the expression of a series of proinflammatory cytokines and chemokines such as IL-1β, IL-6, IL-12, IL-23, and TNF-α; 2) anti-inflammatory M2 macrophages, which are polarized by Th2 cytokines such as IL-4 and IL-13 and dampen inflammation by expressing and releasing anti-inflammatory cytokines such as IL-10 and TGF-β 57. M1 and M2 macrophages have different transcriptional profiles and differentially affect health and disease.

Recently, macrophage epigenome profiling has revealed thousands of chromosomal loci that are differentially active in macrophages, revealing chromosome elements that drive macrophage gene expression. Nucleosomal histone acetylation is one of the most dynamic epigenomic marker. This marker is found at gene promoters and enhancers and correlates adequately with gene expression changes. Imbalance of acetylation and deacetylation in macrophages could result in numerous pathological conditions, such as the development of chronic obstructive pulmonary disease (COPD) 58-60, asthma 61, smoking-induced pro-tumor effect 62 or inflammation 63, 64, sterile inflammation and diabetes comorbidities 65, viral immune evasion 66, disruption of intestinal immune homeostasis 67, and chronic inflammatory responses during atherogenesis 68. HDACi are promising therapeutic agents for these macrophage-related diseases.

Compared to infection with the histone deacetylase-like enzyme (Gc-HDAC)-deficient mutant, higher H3K9ac enrichment at the promoters of proinflammatory mediator genes, various TLRs, adaptor proteins, and transcription factors were detected after infection with *Neisseria gonorrhoeae* 69. The transcription factor ATF-2 correlates with p38, which is associated with stress-induced apoptosis and Toll-like receptor (TLR)-mediated signaling. ATF-2 and LPS synergistically improved the transcription of the Socs-3 promoter. However, HDAC1 could barely interact with ATF-2 after LPS treatment. Treating RAW264.7 cells with trichostatin A, an HDACi, triggered a decrease in LPS-induced Socs-3 expression. This result indicates that HDAC1 promotes Socs-3 transcription after LPS induction in TLR-mediated transcriptional control in macrophage cells 70. In another study, HDACi increased mice vulnerability to bacterial and fungal infections while reinforcing defense against toxic and septic shock, identifying an essential role for HDACi in regulating the expression of innate immune genes and host defenses against microbial pathogens 71. Although prolonged HDACi treatment might injure host defense, selective HDACi may contribute to treating acute bacterial infections 72. TSA could essentially affect anti-apoptosis in LPS-treated macrophages, which was supposed to contribute to the protective effect of TSA in a mouse model of cecal ligation and puncture (CLP)-induced lethal sepsis 73. During polymicrobial sepsis, TSA can adjust the phenotypes of macrophages by facilitating autophagy and reducing inflammation 74. HDACi and DNA methyl transferase inhibitor DNMTi synergistically ablated macrophage death mediated by endotoxemia via STAT3-JMJD3 signaling 75. Similarly, in acute lung injury, combination treatment with HDACi and DNMTi reduces inflammation, while M2 macrophages exhibited an evident increase in quantity, which could be a potential treatment for sepsis 76.

The motility of macrophages, which can infiltrate almost all tissues and exert non-specific immunoregulation and other effects, is an irreplaceable element during self-protective inflammation. Butyrate, a noncompetitive, reversible inhibitor of HDACs, was able to reduce LPS-mediated macrophage migration by suppressing Src and focal adhesion kinase activity 77. TSA was demonstrated to downregulate MMP-9 the expression of in RAW264.7 murine macrophages 78 and reduce macrophage infiltration in tubulointerstitial injury induced by ureteral obstruction 79. Phenylthiobutanoic acids (PTBAs), a new class of HDACi, were able to reduce peritubular macrophage infiltration, thus contributing to recovery and postinjury fibrosis recovery in a progressive mouse model of acute kidney injury 80. Treatment with selective class I HDACi MS-275 could impede macrophage activation and decrease the macrophage infiltration, thus improving the outcome of the cerulein-induced pancreatitis mouse model 81. MGCD0103, a novel orally available and isotype-selective HDACi, could ameliorate macrophage infiltration in a streptozotocin-induced type I diabetes model and thus protects the pancreas from oxidative stress and cell death 82. Administration of class I HDAC and HDAC3 inhibitors MS-275 and RGFP966 in Ang II-induced hypertensive mice could reduce systolic blood pressure and thickness of the aortic wall partly by reducing macrophage infiltration 83. Treatment of Ang II-infused ApoE-/- mice abdominal aortic aneurysm (AAA) models with a class I HDACi (MS-275) or a class IIa HDACi (MC-1568) blocked macrophage infiltration and decreased proinflammatory cytokines 84. Notably, a considerable amount of evidence on HDACi-mediated reduction of macrophage infiltration has been elucidated, and this outstanding discovery provides a promising therapeutic target for multiple human diseases.

*In vitro*, HDACi exert both pro- and anti-inflammatory effects on macrophages. The pro- and anti-inflammatory effects of TSA were separable within a particular concentration range, which means that each HDAC may differentially affect macrophages. MS-275 exhibits proinflammatory effects while downregulating certain inflammatory reactions. In contrast, an HDAC6-selective inhibitor, 17a, which possesses anti-inflammatory functions but no proinflammatory properties. Besides, no significant difference in HDAC-dependent gene expression was detected between HDAC6(-/-) macrophages and normal macrophages, implying that HDAC6 is not the only intermediary for the anti-inflammatory effects of 17a 85. TLR or IFN-γ treatment reduced HDAC5 transcriptional levels in several macrophage cell lines, and blocking HDAC5 expression by specific siRNA significantly decreased the production of proinflammatory cytokines 86. TSA can also block LPS-induced expression of NO synthase II in macrophages by reducing C/EBPβ phosphorylation without affecting DNA binding, which facilitates the production of NO and the cytostatic or cytotoxic activity of macrophages 87. VPA can also induce a phenotype switch towards the M2 subtype 88. The anti-rheumatic activities of the class I/IIb and HDAC1-3 inhibitors, SAHA and MS-275, respectively, such as growth arrested in RA synovial fibroblasts and decreased proinflammatory cytokines and NO, were mediated by p21 expression induction and suppressed NF-κB nuclear accumulation 89. Ky-2, a hybrid-compound HDACi, downregulated the expression of proinflammatory cytokines in LPS-driven human monocyte-like THP-1 cells, thus suppressing M1 macrophage polarization 90. KBH-A42, a new HDACi, plays anti-inflammatory roles by suppressing the transcriptional levels of TNF-α and NO in LPS-stimulated RAW264.7 cells and peritoneal macrophages 91.

The confrontation and balance between M1 and M2 macrophages are necessary to appropriately control the inflammatory response and restore organ function. The anti-inflammatory and antioxidant effects of HDACi VPA on lung macrophages were demonstrated in a nitrogen mustard-induced lung injury model, which was accompanied by an increased M2 phenotype and decreased M1 phenotype of macrophages 92. Inhibition of class I/II HDACs or class III HDACs (Sirtuin) potently influences the activation of macrophages derived from the inflamed joints of patients with rheumatoid arthritis by inhibiting the production of proinflammatory factors, such as IL-6 and TNF-α, and inducing macrophage apoptosis 93. TSA can disrupt inflammatory cytokines by reducing the stability of IL-6 mRNA in IL-1β, TNFα, or Toll-like receptor ligands stimulated macrophages, thus suppressing inflammation in rheumatoid arthritis 94. Recent *in vivo* studies have found that HDACi, specifically, deletion of HDAC3, could promote M2 polarization while dampening the activation of M1 macrophages, thus helping treat atherosclerosis 95, 96. One attractive approach for atherosclerosis therapy is to target macrophage-mediated inflammation. EM-conjugated HDACi, which specifically targets cells expressing carboxylesterase-1, such as monocytes and macrophages, limited peritoneal macrophage maturation but had no inhibitory effect on bone marrow-derived macrophages from WT mice; however, it showed limited efficacy in an atherosclerosis model, 97. In the acute myocardial infarction mouse model, SAHA upregulated M2 markers, thereby facilitating ventricular remodeling and functional recovery 98. In another model of skin wounds, TSA significantly induced an improvement in wound closure by increasing the number of CD11b^low^/Ly6C^low^ subsets of macrophages 99. It has been shown that HDAC11 is a negative regulator of LPS-induced IL-10 production in mouse and human macrophages 100. Furthermore, the suppression of IL-10 in imipramine-treated *L. donovani-*infected macrophages 101 was reported to depend on enhanced HDAC11 102. HDAC11 binds to the distal region of the IL-10 promoter, generating a chromatin configuration with less binding of transcription factors, including SP1 and STAT3 100. Interestingly, miR-145 was shown to target the 3' UTR of HDAC11, positively affecting IL-10 transcription, and IFN-γ stimulation was shown to reduce mouse miR-145 levels through the JAK/STAT signaling pathway 103. Additionally, treating murine macrophages with a pan-HDACi called LAQ824 inhibits IL-10 expression and recruits two transcriptional repressors, including HDAC11, to the distal site of the IL-10 promoter 104. This observation may partially explain why, after an LAQ824 treatment, an increase in a more inflammatory macrophage phenotype, such as production of higher levels of proinflammatory cytokines and capacity to restore anergic CD4+ T cells 104, is observed. The generation of APCs with elevated inflammatory characteristics after HDACi treatment has also been observed in other settings. For example, treatment of murine and human DCs and macrophages, and colitis-induced mice with certain HDACIs, was shown to induce secretion of IL-1β from these cells via a previously unknown caspase-1 independent mechanism 105. Although further verification is required, a pharmacological exclusion approach suggests the involvement of HDAC11 in this process 105.

Another subset of innate immune cells consists of myeloid-derived suppressor cells (MDSCs). They are a mixed population of cells with T cell response suppressing ability, and they consist of precursors and various immature stages of DCs, macrophages, and granulocytes such as neutrophils 106. Consistent with its regulatory role in the expression of the anti-inflammatory cytokine IL-10 in LPS-stimulated macrophages, HDAC11 negatively regulated MDSC expansion and function 107.

Besides, HDACi may also contribute essentially to other functional aspects of macrophages. The pan-HDACi TSA has been demonstrated to decrease CD9 expression in murine peritoneal macrophages, which is implicated in macrophage activation, indicating that HDACi could regulate the expression of membrane proteins involved in matrix interaction; this process may partly contribute to macrophage function modification 108.

In conclusion, HDACs are therapeutic targets in many inflammatory disease models. HDACi may exert their regulatory effects on macrophages in various pathological models via multiple mechanisms, including suppression of macrophage activation, promotion of M2 phenotype affinity, decrease release of proinflammatory cytokine release, inhibition or exacerbation of cell apoptosis, and regulation of phagocytosis or recognition capacity during inflammation.

### HDAC and dendritic cells

Dendritic cells (DCs) originate from hematopoietic stem cells and are distinct from other leukocytes. The main function of DCs is to present antigens to T cells and initiate immune responses. Generally, human DCs can be classified into plasmacytoid DCs, which mainly accumulate in the blood and lymphoid tissues and enter the lymph nodes through the blood circulation, and conventional DCs (cDCs), which refer to all other DCs and comprise cDC1 and cDC2 109, 110. Plasmacytoid DCs express low levels of MHC-II, costimulatory molecules, and integrin CD11c in the steady state and a smaller number of pattern recognition receptors, including TLR-7 and 9. Once TLRs on plasmacytoid DCs are activated, they can produce numerous type I IFN, chemokines, and cytokines and acquire the ability to present foreign cells. cDCs are found in most lymphoid and nonlymphoid tissues and have a strong capacity to sense and capture antigens, which are then processed and presented to T lymphocytes 111.

**Figure 2:**
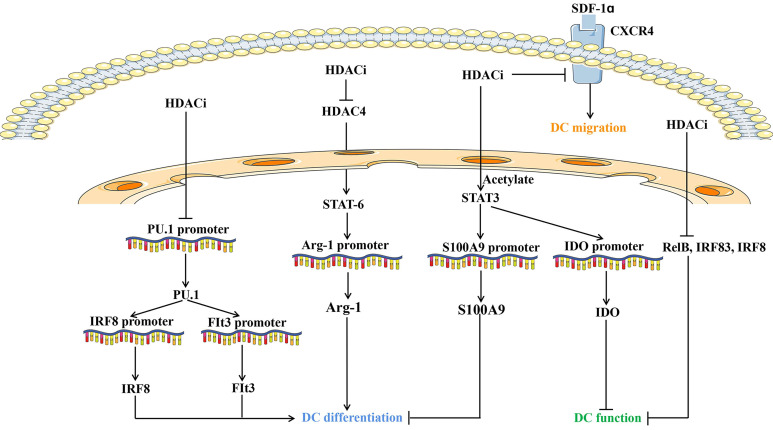
Overview of molecules and pathways implicated in the effect of HDAC inhibitors on dendritic cells development, function and migration. HDACi: HDAC inhibitors; DC: Dendritic cells; Arg1: Arginase 1; SDF-1α: Stromal cell derived factor-1α; IRF: IFN regulatory factor; Flt3: Fms-like tyrosine kinase 3; CXCR4: CXC chemokine receptor 4; IDO: Indoleamine 2,3-dioxygenase.

Interestingly, DC functions are modulated by HDACs (Figure 2). Inhibition of HDAC represses the hallmark gene CD1a and adhesion molecule expression, thus skewing DC differentiation. Defective cytokine secretion, impaired ligand 19-induced migration, and decreased immunostimulatory capacity of DCs upon HDACi exposure appears to prevent signaling through NF-κB, IRF-3, and IRF-8 112, 113. The transcription of RelB, the NF-κB component required for DC differentiation and maturation, is regulated by chromatin modifiers, including HDAC3 114. HDAC4 cross-talks with STAT6 to regulate the transcription of arginase 1, which is essential during mouse DC differentiation 115. Inhibiting HDAC activity prevents mouse DC differentiation by upregulating STAT-3 and indoleamine 2,3-dioxygenase expression 116. Gene expression profiling revealed that HDACs are required for the induction of PU.1 and IRF8 expression and DC development, which is further supported by HDACi studies using TSA, VPA, and MS-275 117. Emerging studies have suggested that liver X receptors are involved in DC-dependent immune responses. Liver X receptor activation and HDAC inhibition balanced the recruitment of STAT3 to the S100A9 promoter and distinctly modified STAT3 118. Moreover, histone acetylation suppresses the chemotactic activity of DCs via the CXCR4-dependent pathway and inhibition of MAP kinases 119. VPA-treated DCs reduced the capacity to activate Th17 cells under LPS stimulation, which is critical for promoting inflammatory responses 120. Collectively, HDAC mainly regulates the differentiation, antigen presenting capacity, and migration of DCs (Figure 2).

### HDAC and lymphocytes

Lymphocytes include natural killer (NK) cells, T and B cells, lymphoid tissue inducing (LTi) cells, and natural helper (NH) cells, among which T and B cells constitute the major components of the adaptive immune system; NK cells, LTi cells, and NH cells are defined as innate lymphoid cells (ILCs). T lymphocytes are subdivided into 1) CD4^+^ T cells, helper T cells (Th cells), 2) CD4^+^ regulatory T cells (Tregs), and 3) CD8^+^ T cells, which are cytotoxic T cells. These naïve cells are exported to the periphery, circulate through the lymph nodes, and find antigens, leading to the activation and differentiation of T cells into effector T cells and memory T cells. CD4^+^ Th cells further differentiate into several functionally distinct Th effector subsets: Th1 cells can stimulate cell-mediated immune responses against intracellular bacteria by producing and secreting IFN-γ. Th2 cells generate Th2 cytokines, such as IL-4, IL-5, and IL-13, for helminth infection. Th17 cells produce interleukins such as IL-17A, IL-21, and IL-22 and initiate immune responses in response to extracellular bacterial and fungal infections. Other subsets, such as T follicular helper cells, Th9, Th22, and TGM-CSF, also play essential roles in regulating immune responses 121. CD8^+^ T lymphocytes mediate immediate and long-term protection against intracellular pathogens NK cells and consist of two main subsets: the cytotoxic CD56^dim^/CD16^pos^ (CD56^dim^) population, which accounts for 90% of peripheral NK cells, and regulatory CD56^bright^/CD16^neg^ (CD56^br^) NK cell subset, which generates pro-inflammatory cytokines 122-124. LTi cells can secrete the pro-inflammatory cytokines IL-17 and IL-22, whereas NH cells can generate large numbers of Th2-type cytokines, such as IL-5, IL-13, IL-6, and IL-9 125. As described above, T and B cells constitute a major part of the adaptive immune system, and ILCs represent another branch of innate immune cells, aside from myeloid cells. Activated B cells, generated from mature B cells, are also known as plasma cells and specialize in the production and secretion of immunoglobulins. Interactions between the innate and adaptive immune systems are crucial for an appropriate immune response against invading pathogens, which requires precise genetic and epigenetic regulation.

HDACi has been demonstrated to regulate the differentiation and function of multiple subsets of CD4 and CD8 T cells 126. The RORγT gene encodes a Th17 lymphocyte signature transcription factor. The differentiation stage-specific effect of HDACi-mediated H4 acetylation by butyrate and apicidin is involved in epigenetic regulation of RORγT expression in human Th17 cells, suggesting the therapeutic potential of HDACi for diseases in which Th17 cells play vital roles 127. TSA was shown to improve allogeneic islet transplantation in diabetic C57BL/6 mice by enhancing Treg cells while suppressing Th17 cells 128. Moreover, in a unilateral ureteral obstruction mouse model, TSA decreased the conversion of Tregs into inflammation-associated IL-17+ Tregs, thus attenuating the degree of fibrosis 129. Similarly, a specific class I and II HDAC inhibitor (ITF2357) decreased the proportion of Th17 cells while increasing Tregs in a dose-dependent manner and acetylating Foxp3 in an NZB/W mice model, indicating its potential to relieve systemic lupus erythematosus (SLE) 130. The studies described above demonstrated the crucial effect of HDACi in reducing the Th17/Treg ratio, thus releasing immune responses in T cell-associated diseases. HDACs mediate the suppressive effect of delphinidin on the proliferation, differentiation, and activation of T lymphocytes towards Th1, Th17, and Treg in patients with metabolic syndrome showing cardiovascular risks 131.

A recent functional study showed that the expression level of HDAC2 correlates linearly with muscle power improvement, indicating that the baseline level of HDAC2 in peripheral blood mononuclear cells is a good biomarker for the steroid response in patients with Duchenne muscular dystrophy 132. The HDAC2-STAT4 pathway can also reverse Th1 shift-mediated neuronal and sensorimotor functional damage, as revealed in an ischemic stroke mouse model treated with antagomir-494 133. Specific HDAC6 inhibition by ACY-738 reduces SLE pathogenesis in NZB/W mice by increasing the Treg phenotype and altering the early stage of B cell development, thus decreasing the overproduction of autoimmune antibodies 134. Another study confirmed that a novel HDAC6 inhibitor, CKD-L, exerts therapeutic effects on collagen-induced arthritis *in vivo* and rheumatoid arthritis *in vitro* by increasing the immunosuppressive function and inducing the expression of cytotoxic T-lymphocyte associated protein-4 in Treg cells 135. Other HDAC6-selective inhibitors such as ACY-1215 (ricolinostat) and ACY-241 (citarinostat) limited melanoma progression by reducing the production of Th2 cytokines and augmenting T-cell immune properties, providing a promising rationale for translational medicine 136. In addition, ACY-1215 suppressed effector T cell maturation and regulated the functions of CD8^+^ T cells in murine CD8^+^ T cell-related skin inflammation models 137. One approach for the management of chronic obstructive pulmonary disease (COPD) is combining theophylline with low-dose cyclosporine A to reduce inflammation. Silencing of lymphocytes in COPD is correlated with the loss of HDAC2 in CD28nullCD8+ T and NKT-like cells 138. CD8^+^ T lymphocytes can inhibit viral replication by suppressing HIV-1 gene expression in HIV-1-infected individuals However, this capability is impaired after blocking of HDACs, demonstrating that HDACs control the expression of some suppressive factors 139. An effective adaptive immune response against microbial infection requires activation of heterogeneous T lymphocytes. Increased activity of HDAC7, 9, and 11 and decreased HAT of memory T lymphocytes were involved in the protective effect of You-Gui pills in asthma, which is airway inflammation mediated by eosinophilic cells 140. HDAC11 has been shown to be involved in the regulation of different aspects of T cell biology. A recent study showed that HDAC11 is responsible for controlling the acetylation status of T-bet and Eomes gene promoters in naïve T cells and that it is rapidly removed from these promoters after T cell activation 141. Furthermore, HDAC11-deficient effector memory mouse T cells exhibited a more inflammatory phenotype and were less responsive to suppression by Tregs 141. An *in vivo* study revealed more rapid and potent development of graft versus host disease (GVHD) after allograft transplantation of T cells lacking HDAC11 141. These findings indicate that similar to its role in neutrophils, HDAC11 is a negative regulator of inflammatory T cell responses. Furthermore, exacerbation of GVHD upon transfer of T cells lacking HDAC11 may partially explain why not all HDACi have beneficial effects on allograft models. Although vorinostat, an inhibitor of class I and class II HDACs, is effective both in animal models and patients 142-144, treatment with an HDACi with potency against HDAC11, including trichostatin A or panobinostat, was reported to accelerate GVHD by increasing proinflammatory cytokine production 145, 146. HDAC11-deficient T cells not only accelerate GVHD, but also may provide a better anti-tumor response when considering their elevated inflammatory capabilities. In line with this prediction, adoptive transfer of HDAC11-deficient T cells into a syngeneic lymphoma tumor model generated a better anti-tumor response than WT T cells 141. Furthermore, silencing of HDAC11 in Hodgkin lymphoma cells induces expression of OX40 ligand, which is known to be important for the development of an efficient anti-tumor response 147-149, and proinflammatory cytokines, including TNF-ɑ and IL-17, which generate an overall favorable anti-tumor response with more effector and fewer regulatory T cells 150.

Currently, surgery is the only effective clinical management for abdominal aortic aneurysms (AAAs). Gene expression analysis showed that HDAC is deregulated in human AAA, and treatment of an AAA mouse model with MS-275 (class I HDACi) or MC-1568 (class IIa HDACi), which decreases lymphocyte and macrophage infiltration, limited aneurysm expansion 151. HDACi is a promising pharmacotherapy for overcoming human aneurysm progression 84. Activation of HDAC7 was demonstrated to mediate Flagellin-triggered IgE expression in B cells, which initiates food allergy 152.

Based on the above studies, the regulatory effects of HDAC and HDACi on lymphocytes include altering their differentiation and conversion, modulating the intensity of immune responses, and reducing cell infiltration.

### HDAC and innate lymphoid cells

Innate lymphoid cells (ILCs) play key roles in host defense, barrier integrity, and homeostasis, and mirror adaptive CD4^+^ Th cell subtypes in both the usage of effector molecules and transcription factors. Chromatin in proximity to effector genes is selectively accessible in ILCs prior to high-level transcription upon activation. Accessibility of these regions is acquired during development and shows only minor changes after activation. In contrast, dramatic chromatin remodeling occurred in naïve CD4^+^ T cells during Th cell differentiation in a type-2-infection model. This alteration resulted in a substantial convergence of Th2 cells toward ILC2 regulomes. These data indicate extensive sharing of regulatory circuitry across the innate and adaptive compartments of the immune system, despite their divergent development pathways 153.

A recent study demonstrated that the HDACi butyrate suppresses type 2 cytokine production in mouse and human ILC2s and inhibits the proliferation and GATA3 expression of mouse ILC2s. Moreover, butyrate suppresses IL-33- and Alternaria-induced lung inflammation 154. This suppressive effect depends on the HDAC inhibitory effect, rather than on GPR41 or GPR43 154. Similarly, TSA suppressed ILC2-mediated lung inflammation induced by IL-33 and Alternaria 49.

### HDAC and microglia

Microglia, the major resident immune cells in the CNS, are recognized as the brain-specific macrophage population and genetically differ from circulating myeloid cells. Like macrophages in the periphery, they are the most multifunctional and plastic CNS inhabitants and can dynamically adapt to their ever-changing environments. They can also be divided into M1 pro-inflammatory and M2 anti-inflammatory subtypes. M1 microglia may exacerbate neuronal damage by releasing inflammatory cytokines such as TNF-α and nitric oxide and impairing axon regeneration after injury. M2 microglia are healthier cells with enhanced phagocytic activity and a reduced production of inflammatory mediators, and can promote the survival of cortical neurons under both normal and ischemic/hypoxic conditions.

The regulatory effect of HDACi on microglial activation has been revealed in multiple previous studies. LPS-activated microglia exhibited histone hyperacetylation 155, and HDAC2 protein levels were significantly upregulated in microglia in the substantia nigra of Parkinson's disease compared to normal controls 156, indicating the regulatory effect of HDACs on microglia in neuroinflammation. *In vivo* studies demonstrated that VPA or NaBu treatment decreased the number of microglia in the ischemic brain, which contributed to reduced brain infarct volume and neurological deficits in a rat model of permanent middle cerebral artery occlusion 157, 158. NaBu administration in a similar model significantly downregulated the expression of TNF-α and NOS2 and upregulated IL-10 expression in activated microglia, thus alleviating neuroinflammation 159. In a traumatic brain injury model, administration of a novel HDAC inhibitor, DMA-PB, reduced the density of phagocytic microglia 160. In a spinal cord injury (SCI) model, injection of VPA attenuated the accumulation of microglia in the injured spinal cord and thus contributed to reducing gliosis after SCI 161. VPA decreased the number of activated microglia and mRNA levels of pro-inflammatory marker genes in an LRRK2 R1441G transgenic Parkinson's disease mouse model 162. In an APP/PS1 transgenic mouse model of Alzheimer's disease, inhibition of HDAC3 in the hippocampus alleviated microglial activation, which showed therapeutic potential for Alzheimer's disease 163. MS-275 alleviates postoperative cognitive dysfunction by reducing the number of activated microglia and neuroinflammation in the hippocampus of rats via HDAC inhibition pretreatment with MS-275 164. The anti-neuroinflammatory effect of HDACi has been demonstrated in various animal disease models, providing a basis for related clinical transformation.

However, *in vitro* studies have shown contradictory results. The HDAC inhibitors TSA, CAY10683, apicidin, MS-275, and MI192 reduced the expression of inflammatory mediators such as IL-6, IL-1β, and TNF-α in LPS-treated BV-2 microglial cells 165-167. VPA, TSA, and NaBu can induce apoptosis of microglia cells, and pretreatment with sodium butylate or TSA in mesencephalic neuron-glia cultures led to a decrease in LPS-induced pro-inflammatory responses and damage to dopaminergic neurons 168. TSA administration in BV-2 and primary microglia reversed the suppressive effect of LPS on *Rgs10* transcription, which was able to regulate the production of pro-inflammatory cytokines in microglia 169. In a rat model of SCI, VPA treatment also promoted the differentiation of microglia into the anti-inflammatory M2 subtype by inhibiting HDAC3 and subsequent STAT1-mediated acetylation of the NF-κB pathway 170. SAHA treatment in LPS-stimulated glial cultures was found to strengthen the immunosuppressive effects of dexamethasone 171. In addition, Scriptaid, a novel HDACi, stimulated microglia to switch to the M2 phenotype. This phenotypic switch relieved the inflammatory response in a mouse model of traumatic brain injury 172. In a neonatal rat model of hypoxia-ischemia, NaBu treatment exerted a similar effect as Scriptaid by promoting neurogenesis in the subventricular zone 173. The transition effect of sodium butylate also reversed the proliferative and neurotoxic effects of microglia mediated by Runt-related transcription factor 1, translocated to 1, which was reported to interact with HDACs and is involved in epigenetic modification of Cdk4 and LAT2 174.

After LPS treatment, however, the expression level of HDACs was increased in both primary microglia cultures and acutely isolated microglia from LPS-treated mice, and HDACi TSA and SAHA strongly suppressed LPS-induced cytokine release by microglia. Furthermore, the expression of M1- and M2-associated activation markers was suppressed and the migratory behavior of microglia was attenuated 175. Similarly, TSA and SAHA treatment in LPS-activated murine N9 and primary microglia increased the expression of pro-inflammatory cytokines such as IL-6, TNF-α, and nitric oxide 176, suggesting a pro-inflammatory effect for HDACi. VPA and NaBut increased the release of pro-inflammatory cytokines in activated microglia 177. The variations among different studies may be related to differences in the doses of administration and between models used *in vivo* and *in vitro*.

Thus, although there are some contradictory results, the anti-inflammatory effect of HDAC on microglia has been demonstrated in most related studies and HDAC may function by reducing the number of microglia, generating cytokines, and promoting the phenotype switch towards M2.

### HDAC and astrocytes

As the largest group of glial cells in the CNS, astrocytes play major roles in various manners, such as by maintaining blood-brain barrier integrity, wiring neural circuitry, regulating metabolism, and neuroinflammation, and when pathologically activated, further undergoing a series of morphological and functional adaptations, including proliferation, to form a barrier that separates the injury site and by releasing cytokines and chemokines 178. These reactive astrocytes can be classified into the A1 and A2 anti-inflammatory subtypes 179, 180. A1-reactive astrocytes are thought to be harmful to neurons and oligodendroglia *in vivo*. In contrast, A2-reactive astrocytes promote neuronal survival and tissue repair by upregulating neurotrophic factors.

Epigenetic regulation by HDAC can coordinate astrocyte function by affecting activation and cytokine production. It has been demonstrated that reduced HDAC levels in astrocytes by dimethyl fumarate treatment contribute to the suppression of inflammatory responses 181. In addition, the mood-stabilizing and anticonvulsant drug VPA modulated the mRNA levels of neuroligin-1 and neuregulin-1, which function in post-synaptic cell adhesion, and two extracellular matrix proteins (neuronal pentraxin-1 and thrombospondin-3) in astrocytes to balance excitatory/inhibitory synapse formation in neuro-glial mixed cultures 182. In LPS-or 1-methyl-4-phenylpyridinium-treated neuron-glia cultures, VPA, TSA, and NaBu protected dopaminergic neurons by upregulating glial cell line-derived neurotrophic factor and brain-derived neurotrophic factor expression in astrocytes 183, 184. Moreover, VPA, sodium butylate, TSA, MS-275, and apicidin recruit the transcription co-activator p300 and upregulate H3K4me2 modification at the HSP70 promoter, enhancing neuroprotective HSP70 expression in rat astrocytes 185. M344, a specific HDAC 1/6 inhibitor, and VPA increased acetylated glutamate carboxypeptidase II (GCPII) protein and decreased the GCPII ubiquitination level. In contrast, C646, a histone acetyltransferase inhibitor of p300/CBP, significantly reduced the level of GCPII protein. Therefore, the increase in GCPII induced by VPA does not occur via a classical epigenetic mechanism, but rather by enhanced acetylation of lysine residues in GCPII 186. A highly selective HDAC3 inhibitor, RGFP966, downregulates astrocyte activation, showing protective effects against huntingtin-induced neuronal toxicity and cell death in an N171-82Q transgenic mouse model of huntingtin 187. These results demonstrate that HDACi-treated astrocytes exert neuroprotective effects in diverse manner. Moreover, although there is emerging evidence that astrocytes exhibit regional heterogeneity, whether HDACs control this diversity remains unknown.

### HDAC and hematological malignant cells

In addition to regulating the biological activities of immune cells, HDACs play a role in several hematological pathologies (Table 2). Therefore, HDACi have been gradually applied in the clinical treatment of bone marrow failure and hematopoietic malignancies. For example, combining immunotherapy with HDACi helps overcome immunotherapy resistance in B-cell lymphoma 188.

Expression of HDAC1, HDAC2, and HDAC6 was significantly upregulated in PTCL and diffuse large B-cell lymphoma, and HDAC6 levels indicate a favorable prognosis 189. In Hodgkin's lymphoma, HDAC1, 2, and 3 are highly expressed, whereas low levels of HDAC1 may be a marker of worse outcomes 190. HDAC activity in CD19^+^ B cells purified from patients with chronic lymphocytic leukemia was found to be negatively correlated with treatment-free survival and overall survival 191. The expression of HDACs is enhanced in NK cells from both aggressive and chronic NK leukemia 192. The transcription of HDAC3 was repressed by the chimeric transcription factor PML-RARalphain, which leads to the development of acute promyelocytic leukemia 193.

HDACi can target hematological malignant cells. Histone deacetylation has been shown to downregulate Bcl-6 activity, and TSA treatment can accumulate inactive acetylated Bcl-6, thus leading to cell cycle arrest and apoptosis of B-cell lymphoma cells 194. Similarly, TSA exerted anti-cancer effects on Epstein-Barr virus-transformed lymphoblastoid cell lines by inducing cell cycle arrest, apoptosis, and the Epstein-Barr virus lytic cycle 195. Addition of TSA to a cytokine cocktail containing TNF-α, GM-CSF, and ckit ligand significantly enhanced the treatment effects to promote the differentiation of acute myeloid leukemia and acute lymphoblastic leukemia cell lines towards DCs 196. In another study, HDACi TSA, *n*-butyrate, and apicidin induced the differentiation of the eosinophilic leukemia EoL-1 cell line into eosinophils 197. SAHA arrests the cell cycle, decreases cell survival, and induces differentiation of acute myeloid leukemia and myelodysplastic syndrome cell lines 198. SAHA also strengthened the cytotoxic effects of cladribine towards leukemic NK cells through intrinsic mitochondrial and extrinsic death receptor pathways 192. MS-275 arrests the cell cycle at low concentrations while inducing apoptosis at higher concentrations in several human leukemia and lymphoma cell lines 199. Similarly, butyrate likely inhibits the proliferation and increases the apoptosis of mastocytoma P815 cells 200, indicating the anti-cancer capacity of HDACi and providing insight into their clinical transformation. AR-42, a novel HDACi, induced growth inhibition, cell cycle arrest, apoptosis, and activation of caspases-3/7 in malignant mast cell lines 201. Additionally, LBH-589 inhibits multiple myeloma cell growth by degrading the catalytic subunit of calcineurin, PPP3CA. This degradation may be mediated by suppression of the chaperone function of HSP90 due to HDAC6 inhibition 202.

## Conclusion and Perspective

**Figure 3:**
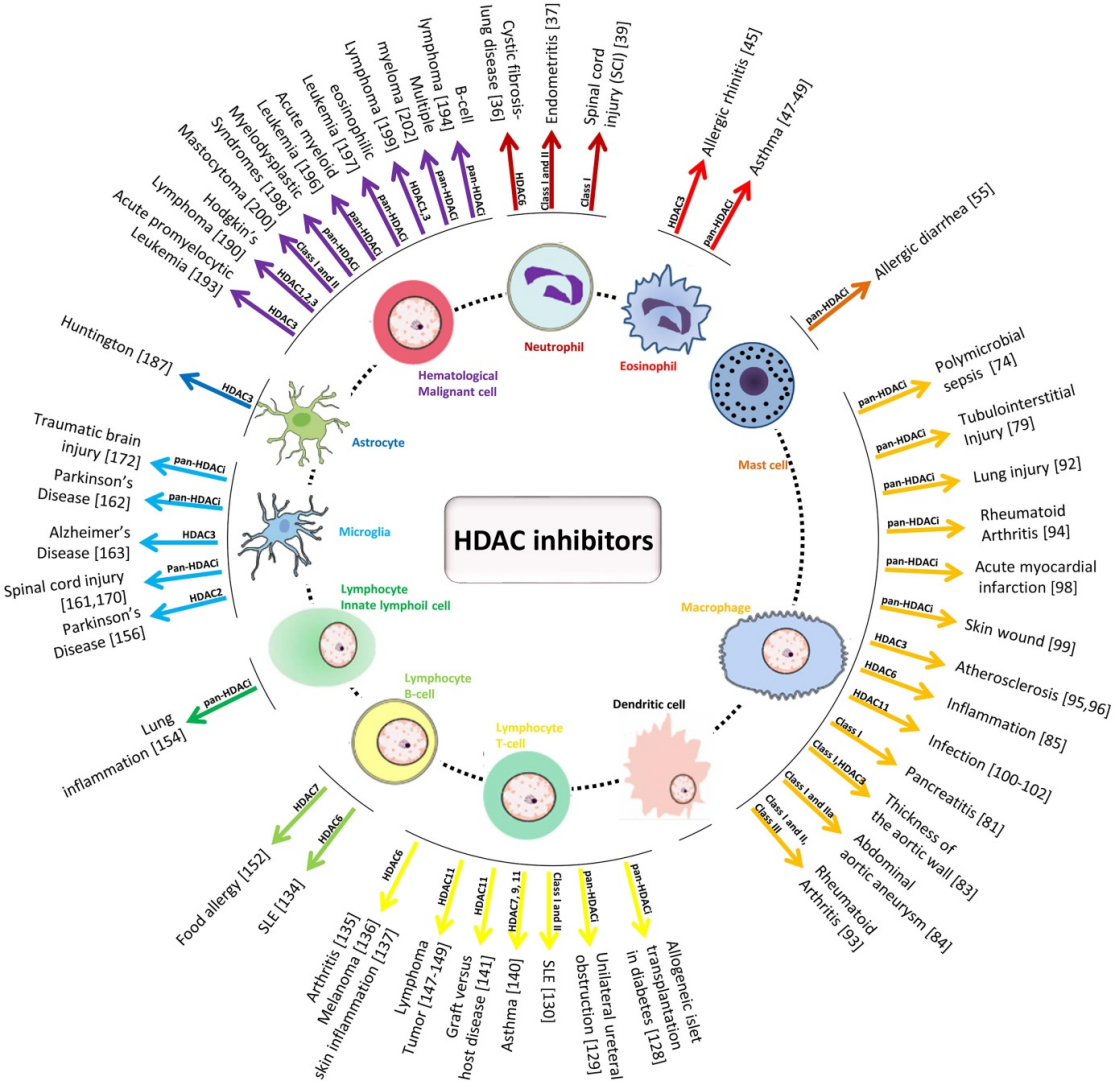
Schematic diagram of the effect of HDAC inhibitors on immune cells and the detailed HDACs involved in different inflammation-related diseases.

In this review, we summarized the results of preclinical investigations of the regulatory effects of HDACs and HDACi on immune cells in related diseases (Figure 3). As illustrated, there are several ways by which HDAC and HDACi modulate the development and functions of immune cells, affect the proliferation and differentiation of hematopoietic stem and precursor cells, and regulate inflammatory responses by adapting the phenotype switch of mature immune cells, inducing or inhibiting the apoptosis of effective immune cells and their infiltration into inflammatory sites.

**Figure 4:**
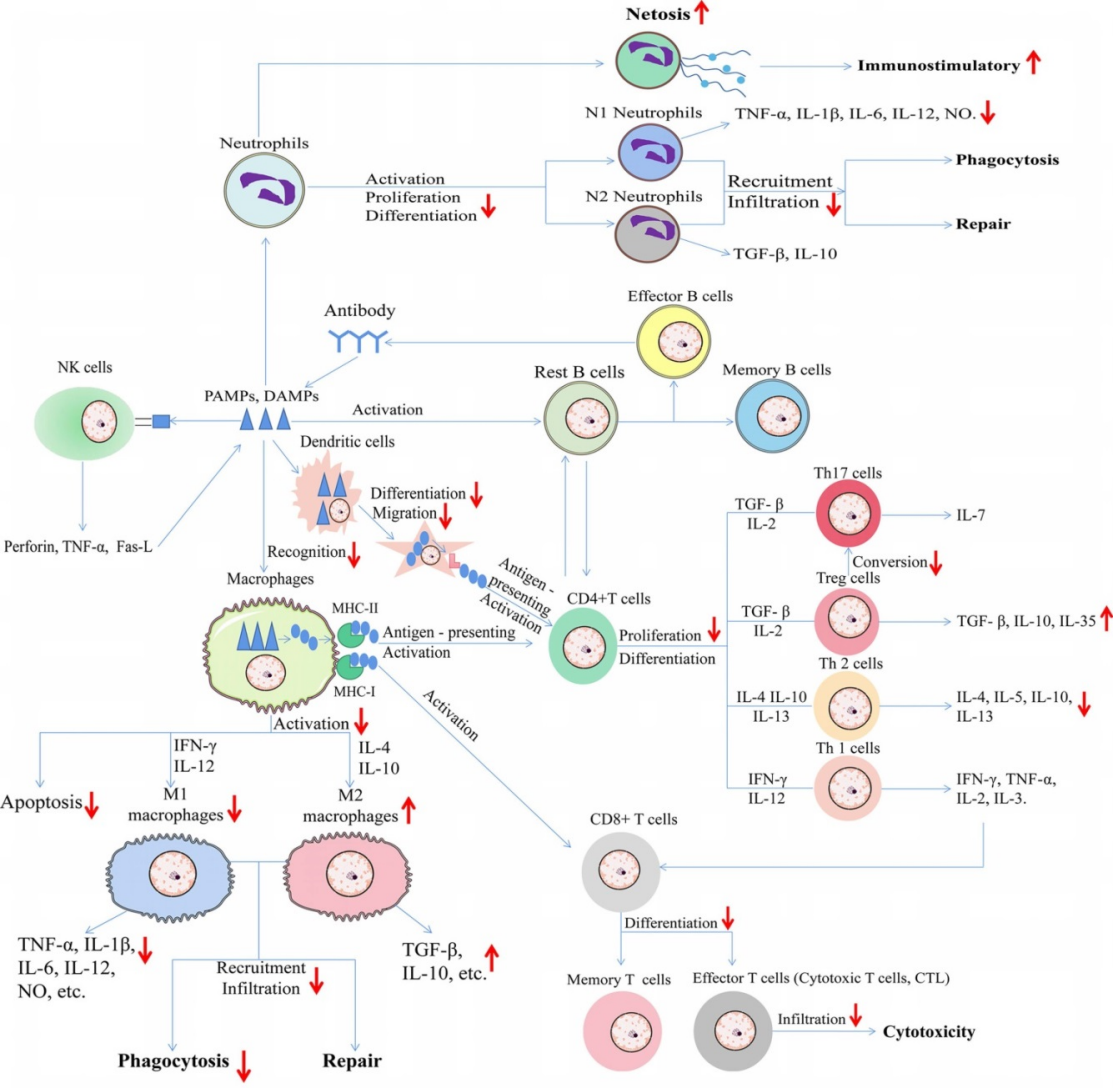
Schematic diagram of effect of HDAC inhibitors on immune cells during immune responses to diverse pathogen-associated molecular patterns (*PAMPs*) from microorganisms as well as damage-associated molecular patterns (*DAMPs*) from dead or dying host cells. The upward arrows represent the promotion effect by HDAC inhibitors and the downward arrows represent the suppression effect by HDAC inhibitors. TNF-α: Tumor necrosis factor-α; IL: Interleukin; NO: Nitric oxide; TGF-β: Transforming growth factor; IFN-γ: Interferon-γ; MHC: major histocompatibility complex; Fas-L: Fas ligand.

In particular, under certain pathological conditions, different immune cells interact with each other. In one hand, we summed up the effect of HDACi on immune cells during type 1 allergy (Figure 1). In another hand, we summerize the network diagram of cell-cell interaction, as well as the effect of HDACi on immune cells during immune responses to diverse pathogen-associated molecular patterns (PAMPs) from microorganisms as well as damage-associated molecular patterns (DAMPs) from dead or dying host cells (Figure 4).

HDACi treatment decreases inflammatory responses in most cases and can alleviate symptoms in animal models of autoimmune and inflammatory diseases. Notably, HDACi show bidirectional regulatory effects on immune responses; for example, pretreatment with TSA relieved the inflammatory responses of microglia in the mouse brain after LPS injection 165, whereas TSA aggravated the inflammatory response when added at the same time as LPS *in vitro* 176. HDACi have been shown to suppress overactivated autoimmune responses 135, 137, although in other studies, HDACi enhanced the cytotoxic effect of lymphocytes in malignant tumors 136 and HIV-1 139. These results suggest that the regulatory role of HDACi in immune cells is phase-and context-dependent. Selective inhibition of HDAC6 exerted different functions in different immune cells. The mechanism underlying this biphasic role remains unclear, and further fundamental research is needed to improve the use of HDACi in immune-related diseases.

Moreover, there are concerns regarding the effectiveness and safety of treating other diseases, although the therapeutic benefit of HDACi on hematological malignancies is unclear. For example, most currently used HDACi are non-selective pan-HDACi, and their relatively low specificity may alter the expression of thousands of vital genes, thus leading to undesirable consequences and impeding the widespread clinical application of HDACi. Therefore, the specificity of these molecular compounds should be further improved and toxicity should be carefully controlled. VPA, a widely prescribed antiepileptic drug, is known to induce hepatotoxicity. To date, selective HDACi have been modified in several regions 203-205. However, because of the similarity among the crystal structures of different HDACs, their selectivity remains unclear, as most current 'selective' inhibitors may actually bind to multiple HDAC isoforms 206. Thus, more accurate knowledge of the crystal structures of HDACs and HDACi is required to develop more sophisticated and better modified HDACi to match the subtle differences among different HDAC isozymes.

Researchers currently have a general understanding of how epigenetic modulation by HDAC and HDACi regulates the development and function of immune cells. HDACi may serve as epigenetic modifiers, which have been explored as single agents and in combination with other established therapies and can reverse some pathological conditions. In future research, more efforts should be focused on the issues of specificity to broaden the field of clinical application, which will open new pathways for pharmacological interventions in multiple related diseases.

## Figures and Tables

**Table 1:** The chemical structures and targets of HDAC inhibitors

Structure	Chemical Name (Abbreviation)	Targets
Short-chain fatty acids	Valproic acid [VPA]	pan-HDACi
	Sodium butyrate [NaBu]	class I and II HDACi
Benzamides	DMA-PB	pan-HDACi
	Entinostat [MS-275]	class I HDACi
	Mocetinostat [MGCD0103]	class I HDACi
	MI192	selective HDAC2/3 inhibitor
	M344	selective HDAC6-inhibitor
Hydroxamic acids	Panobinostat [LBH-589]	pan-HDACi
	Belinostat [PXD101]	pan-HDACi
	Vorinostat [SAHA]	pan-HDACi
	Trichostatin-A [TSA]	pan-HDACi
	Dacinostat [LAQ824]	pan-HDACi
	AR-42	pan-HDACi
	Panobinostat [LBH-589]	pan-HDACi
	Ky-2	pan-HDACi
	Scriptaid [ST]	class I HDACi
	Givinostat [ITF2357]	class I and II HDACi
	MC-1568	class IIa HDACi
	17a	selective HDAC6-inhibitor
	Santacruzamate A [CAY10683]	selective HDAC2-inhibitor
	Tubacin	selective HDAC6-inhibitor
	PTG-0861	selective HDAC6-inhibitor
	KA2507	selective HDAC6-inhibitor
	Citarinostat [ACY-241]	selective HDAC6-inhibitor
	ACY-738	selective HDAC6-inhibitor
	ACY-1215 (ricolinostat)	selective HDAC6-inhibitor
	ACY-241 (citarinostat)	selective HDAC6-inhibitor
	Ricolinostat [ACY-1215]	selective HDAC6-inhibitor
	CKD-L	selective HDAC6-inhibitor
Cyclic peptides	Apicidin	pan-HDACi
	KBH-A42	pan-HDACi
	Romidepsin [FK228]	selective HDAC1/2-inhibitor
Miscellaneous compounds	Depudecin	pan-HDACi
	RGFP966	selective HDAC3-inhibitor

**Table 2:** Inhibition of HDAC6 inhibitors on immune cells function in different disease models

Inhibitor Name	Disease Models	Immune cells	Effect of HDAC6 inhibitors	References
HDAC6(-/-)	LPS	Macrophages	Showed normal LPS-induced expression of HDAC-dependent inflammatory genes	85
HDAC6-shRNAs, or Tubacin	Cystic fibrosis-lung disease	Neutrophils	Reduce the release of IL-6 and MPO	36
ACY-738	SLE pathogenesis	T-cell; B-cell	Increase the Treg phenotype and alter the early stage of B cell development thus decrease the overproduction of autoimmune antibody	134
CKD-L	Collagen-induced arthritis	T-cell	Increase the suppressive function of Treg cells	135
ACY-1215, ACY-241	Melanoma	T-cell	Decreased Th2 cytokine production, augmented T-cell immune properties	136
ACY-1215	Skin inflammation models	T-cell	Suppress the generation of effector T cells from naive CD8+T cells and regulating the activation and functions of CD8+T cells	137

**Table 3:** HDAC inhibitors and their anti-tumor effect in hematological malignant cells

HDAC inhibitors	Hematological malignant cells	References
TSA	B lymphoma cell line	194
	Lymphoblastoid cell line	195
	Myeloid leukemia (AML) cell line	196
	Acute lymphoblastic leukemia cell line	196
	Eosinophilic leukemia EoL-1 cell line	197
MS-275	Leukemia and lymphoma cell lines	199
Vorinostat (SAHA)	Myeloid leukaemia (AML) cell line	198
	Myelodysplastic syndromes cell line	198
	Leukaemic NK cells	192
AR-42	Malignant mast cell line	201
Butyrate	Mastocytoma P815 cell line	200
LBH-589	multiple myeloma cell	202
